# Characterization of a prognostic model for lung squamous cell carcinoma based on eight stemness index-related genes

**DOI:** 10.1186/s12890-022-02011-0

**Published:** 2022-06-08

**Authors:** Wenfa Jiang, Ning Xie, Chenyang Xu

**Affiliations:** Thoracic Surgery Department, Ganzhou People’s Hospital, 16 MeiGuan Ave, Zhanggong, 341000 Ganzhou China

**Keywords:** Cancer stem cells, Risk score, Immune checkpoint genes, Tumor-infiltrating immune cells, Risk stratification

## Abstract

**Background:**

Cancer stem cells (CSCs) are implicated in cancer progression, chemoresistance, and poor prognosis; thus, they may be promising therapeutic targets. In this study, we aimed to investigate the prognostic application of differentially expressed CSC-related genes in lung squamous cell carcinoma (LUSC).

**Methods:**

The mRNA stemness index (mRNAsi)-related differentially expressed genes (DEGs) in tumors were identified and further categorized by LASSO Cox regression analysis and 1,000-fold cross-validation, followed by the construction of a prognostic score model for risk stratification. The fractions of tumor-infiltrating immune cells and immune checkpoint genes were analyzed in different risk groups.

**Results:**

We found 404 mRNAsi-related DEGs in LUSC, 77 of which were significantly associated with overall survival. An eight-gene prognostic signature (PPP1R27, TLX2, ANKLE1, TIGD3, AMH, KCNK3, FLRT3, and PPBP) was identified and used to construct a risk score model. The TCGA set was dichotomized into two risk groups that differed significantly (*p* = 0.00057) in terms of overall survival time (1, 3, 5-year AUC = 0.830, 0.749, and 0.749, respectively). The model performed well in two independent GEO datasets (*p* = 0.029, 0.033; 1-year AUC = 0747, 0.783; 3-year AUC = 0.746, 0.737; 5-year AUC = 0.706, 0.723). Low-risk patients had markedly increased numbers of CD8+ T cells and M1 macrophages and downregulated immune checkpoint genes compared to the corresponding values in high-risk patients (*p* < 0.05).

**Conclusion:**

A stemness-related prognostic model based on eight prognostic genes in LUSC was developed and validated. The results of this study would have prognostic and therapeutic implications.

**Supplementary Information:**

The online version contains supplementary material available at 10.1186/s12890-022-02011-0.

## Background

Lung cancer is one of the most common and deadliest global malignancies [1]. Lung squamous cell carcinoma (LUSC), a primary subtype of non-small cell lung cancer (NSCLC), accounts for approximately 30% of all lung cancer cases [2]. LUSC is more common in men than in women and is largely attributed to smoking habits [3]. Although approximately 70% of stage I patients survive for more than 5 years, the total five-year survival rate of LUSC is roughly 20%, largely because LUSC is often detected at an advanced stage [4, 5].

Cancer stem cells (CSCs) are a subgroup of pluripotent cells possessing a high capability of self-renewal, differentiating into various cell types, and acquiring stem-cell-like features [6, 7]. According to the widely accepted CSC theory, CSCs are implicated in tumor initiation, growth, and metastasis [8, 9]. CSCs are major contributors to the resistance to conventional therapies, tumor recurrence, and poor prognosis. Therefore, targeting CSCs offers a new approach to developing efficient therapies and improving outcomes [10, 11]. An increasing evidence shows that CSCs have prognostic value and could serve as potential prognostic biomarkers in various cancers, including lung cancer [12–14]. Recently, Liao et al*.* identified key cancer stemness-related genes implicated in LUSC through integrated bioinformatics analysis [15]. Similarly, Qin et al. screened LUSC mRNA-related hub genes through bioinformatics and concluded that these genes may serve as therapeutic targets for inhibiting the LUSC stem cell properties [16]. Nevertheless, there is a lack of research on the application of cancer stemness-related genes as prognostic tools in LUSC.

Many studies have been conducted on the derivation of gene signatures as a way of determining prognostic potential; for example, Giannos et al. identified prognostic genetic biomarkers for cell lung cancer progression through comprehensive bioinformatics analysis [17], and Wu et al. identified hub genes and important KEGG pathways closely related to the occurrence and development of lung adenocarcinoma by analyzing gene expression microarrays [18]. In the present study, we used publicly available transcriptomic data and the mRNA expression-based stemness index (mRNAsi) as a quantitative reflector of cancer stemness to screen mRNAsi-related genes with prognostic potential. We then used the results to construct a risk score model for survival prediction in LUSC. Two independent cohorts were used to validate the prognostic performance of the risk score model. The molecular mechanisms underlying the survival subgroups were explored. The results of this analysis would contribute to the subtyping of survival groups and a more refined prognosis of LUSC.

## Methods

### Data source and retrieval

Gene expression data (FPKM value, Illumina HiSeq 2000 platform) from 501 tumor samples and 49 matched normal samples were downloaded from The Cancer Genome Atlas (TCGA) data repository (https://gdc-portal.nci.nih.gov/). Among these, 494 tumor samples with corresponding clinical prognosis information were used as the training set (TCGA set).

We searched for the validation datasets in the NCBI GEO database using lung cancer and *Homo sapiens* as keywords. The inclusion criteria were as follows: histological information available, 150 or more samples, including 50 or more LSCC samples, and overall survival (OS) information of LSCC samples available. Consequently, the GSE30219 [19] (N = 307) and GSE37745 [20, 21] (N = 196) datasets (GPL570 Affymetrix Human Genome U133 Plus 2.0 Array) met the criteria and were used as validation datasets in the current study, containing 58 and 66 LUSC samples, respectively, with corresponding clinical prognosis information.

### Evaluation of mRNAsi scores and differentially expressed genes

Stem cell features of the tumor samples were evaluated using mRNAsi values, which were calculated using a one-class logistic regression machine-learning algorithm in the *gelnet* package in R software (version 2.41-1); the protocol has been described in detail in our previous report [22].

We compared the mRNAsi scores of tumor and normal samples using the t-test in the R software program (version 3.6.1). Based on the median mRNAsi score, the tumor samples were categorized into low- and high-mRNAsi groups (values being below or above the median mRNAsi). Kaplan–Meier (KM) survival curves were plotted for each group and compared using the log-rank test.

We used the *limma* package [23] in R to screen for differentially expressed genes (DEGs), setting the threshold of significance at FDR < 0.05 when comparing tumor and normal samples and |log_2_FC|> 0.5 when comparing low- and high-mRNAsi samples. The genes that were common between the two lists of DEGs were then subjected to the gene ontology (GO) function and Kyoto Encyclopedia of Genes and Genomes (KEGG) pathway enrichment analysis using DAVID software (version 6.8, https://david.ncifcrf.gov/). Statistical significance was set at *P* < 0.05.

### Risk score model for survival prediction

Univariate Cox regression analysis was performed to identify survival-related common DEGs (log-rank *p* value < 0.05) using the *survival* package in R [24]. Of the survival-related genes, prognostic genes were identified by performing L1-penalized least absolute shrinkage and selection operator (LASSO) Cox regression analysis [25] using the *penalized* package. We used 1000-fold cross-validation to determine the optimal lambda value, the penalty parameter, corresponding to the minimal mean-squared error. Consequently, based on a linear combination of the LASSO Cox regression coefficients multiplied by the expression value of each optimal prognostic gene, the risk score was calculated for each sample in the TCGA set using the following formula:

$${\text{Risk score PS}}) = \sum\upbeta _{{{\text{DEGs}}}} \times {\text{Exp}}_{{{\text{DEGs}}}}$$

where β_DEGs_ and Exp_genes_ represent the regression coefficients and expression values, respectively.

Based on the median risk score, the tumor samples were then separated into high- and low-risk samples (having values above or below the median risk score).

### Analysis of tumor-infiltrating immune cells and immune checkpoint genes

Differences in the fractions of tumor-infiltrating immune cells (TIICs) between high- and low-risk samples were analyzed using CIBERSORT [26] software. The expression levels of 14 immune checkpoint genes were also compared between the high- and low-risk samples.

### Pathway enrichment analysis and protein prediction

Based on the gene expression data, we performed the KEGG pathway enrichment analysis using the Gene Set Enrichment Analysis described in the literature [27] (FDR < 0.05). We then accessed the Human Protein Atlas (HPV) database [28] (version 18, https://www.proteinatlas.org/) to search for immunohistological images of the proteins encoded by the risk score genes in tumor and normal tissues.

## Results

### Identification of mRNAsi-related DEGs

**Fig. 1:**
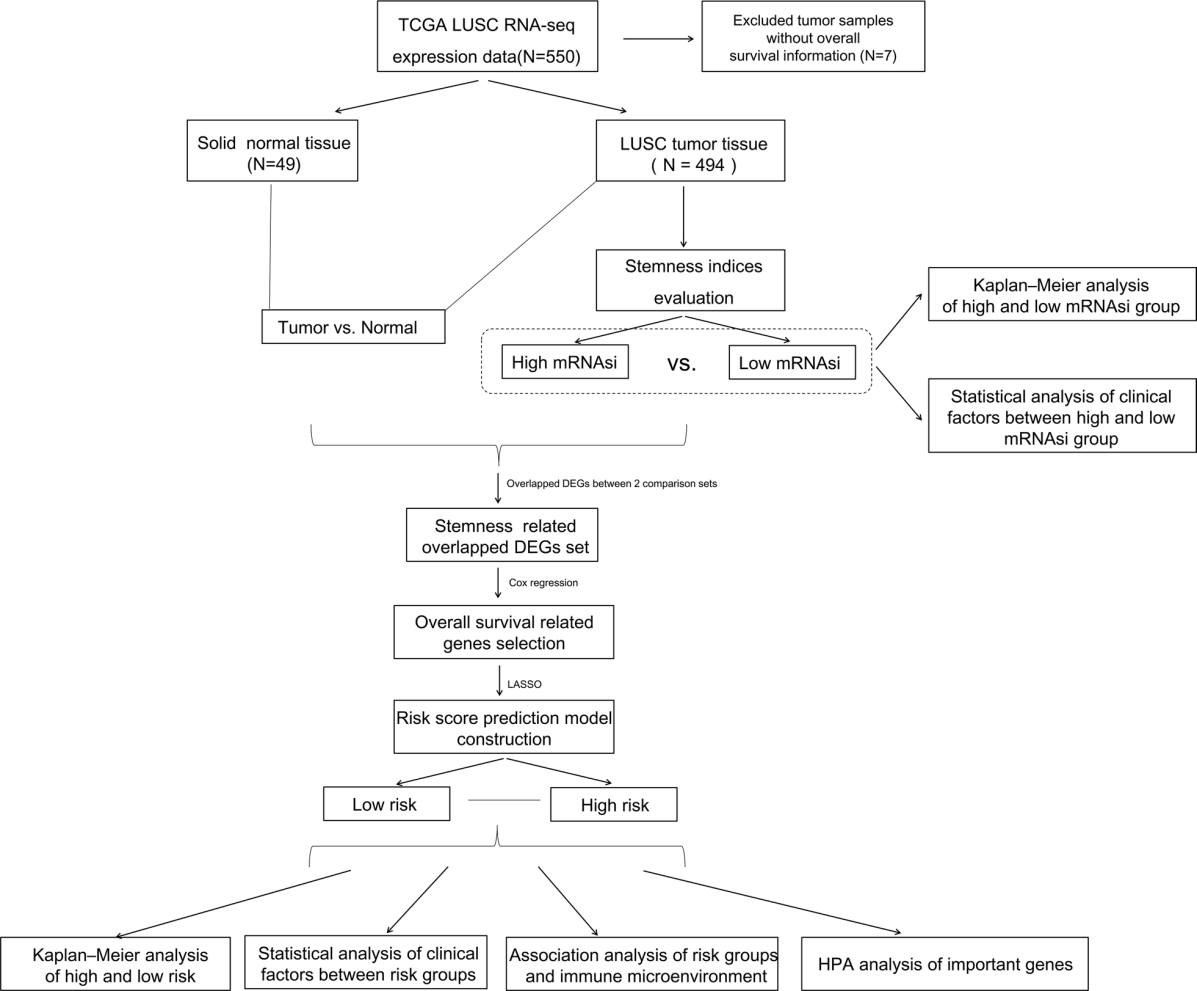
The flow diagram of this study

**Fig. 2:**
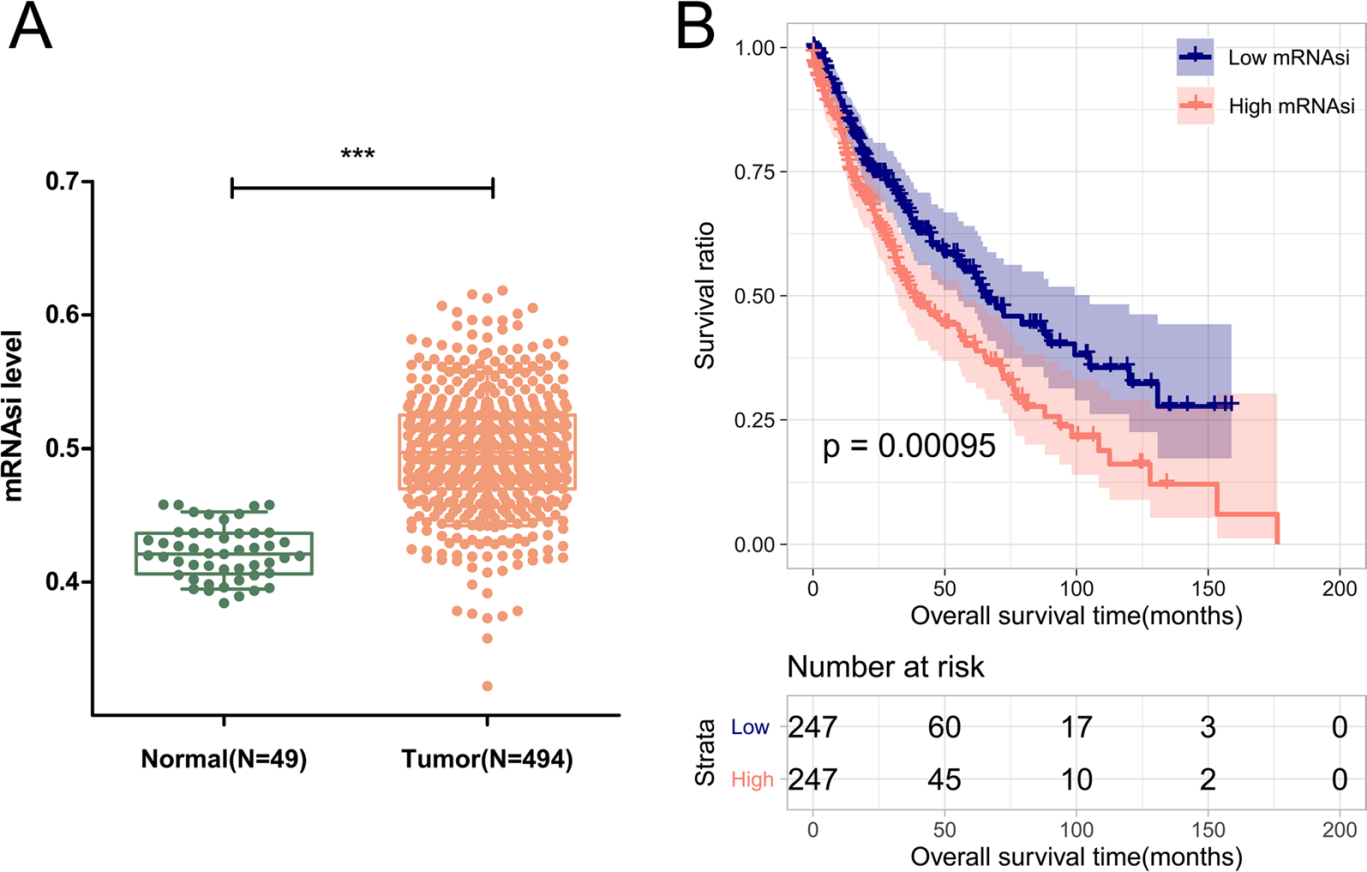
Analysis of mRNA stemness index (si). **a** Different mRNAsi values in normal and tumor tissue; **b** Kaplan–Meier survival curves of high- and low-mRNAsi tumor samples

A flowchart depicting the overall design of the study is shown in Fig. 1. Using TCGA data, we found that mRNAsi was significantly higher in tumor tissues than in normal tissues (*p* < 0.001, Fig. 2a). Upon analyzing the median mRNAsi values, it was found that tumor samples could be categorized into high- and low-mRNAsi groups, with significantly different overall survival (OS) times (*p* = 0.00095, Fig. 2b). Additionally, age was significantly different between the high- and low-mRNAsi samples (*p* = 1.20E−02, Table 1).

**Table 1:** Clinical characteristics of high and low mRNAi samples

Characteristics	N of cases	mRNAsi level		*P*-value
		Low	High	
Age (years)				
≤ 60	107	65	42	1.20E−02
> 60	382	179	203	
Gender				
Male	366	181	185	7.58E−01
Female	128	66	62	
Pathologic M				
M0	406	211	195	2.71E−01
M1	7	2	5	
Pathologic N				
N0	316	151	165	3.70E−01
N1	127	68	59	
N2	40	24	16	
N3	5	2	3	
Pathologic T				
T1	114	48	66	1.39E−01
T2	287	154	133	
T3	70	36	34	
T4	23	9	14	
Pathologic stage				
Stage I	242	116	126	4.85E−01
Stage II	158	82	76	
Stage III	83	45	38	
Stage IV	7	2	5	
Tumor recurrence				
Yes	100	51	49	9.98E−01
No	286	144	142	
Radiotherapy				
Yes	50	31	19	7.19E−02
No	376	181	195	

**Fig. 3:**
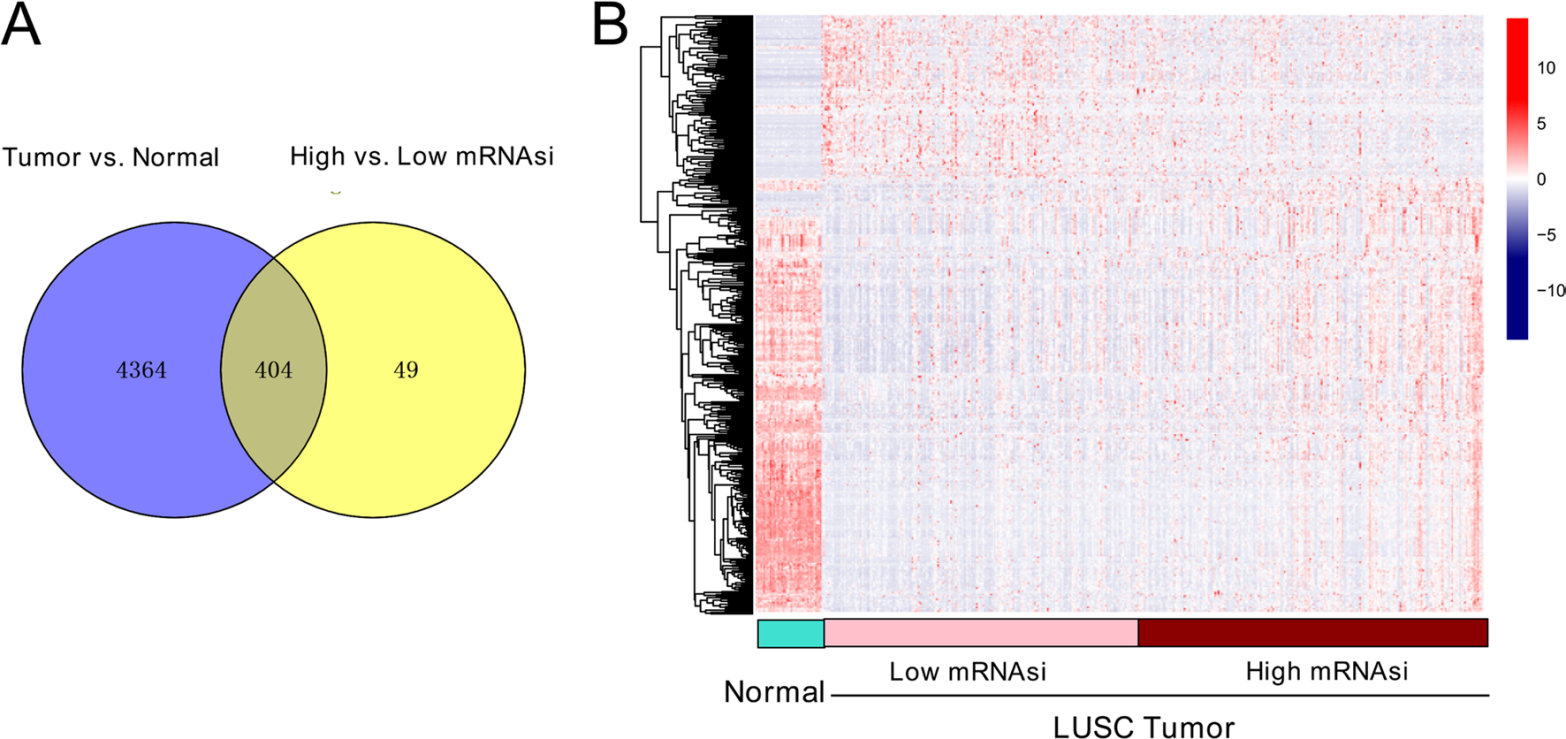
Identification of common differentially expressed genes (DEGs). **a** Venn diagram displaying common genes between the DEGs in tumor and the DEGs between high- and low-mRNAsi tumor samples. **b** heatmap showing expression patterns of the common DEGs in normal samples, low-mRNAsi tumor samples and high-mRNAsi tumor samples

We identified 4,768 DEGs in tumor tissues, compared to normal tissues, and 453 DEGs when comparing high- and low-mRNAsi tumors (FDR < 0.05, |log_2_FC|> 0.5). The two lists of DEGs had 404 common genes (Fig. 3a). Upon clustering analysis, we found that there were significant differences in the expression levels of common DEGs in normal tissues and high- and low-mRNAsi tumor samples (Fig. 3b). A total of 404 mRNAsi-related DEGs were significantly involved in 18 biological processes, including “cell adhesion,” “potassium ion transmembrane transport,” “inflammatory response,” “potassium ion transport,” and “cell–cell signaling” (Table 2). Moreover, these mRNAsi-related DEGs were significantly enriched in 12 KEGG pathways, such as “neuroactive ligand-receptor interaction,” “cAMP signaling pathway,” and “calcium signaling pathway.”

**Table 2:** Summary of significant GO terms and pathways

Category	Term	Count of genes	*P*-value
Gene ontology biology process	GO:0007155 ~ cell adhesion	30	2.19E−07
	GO:0071805 ~ potassium ion transmembrane transport	12	5.88E−5
	GO:0006954 ~ inflammatory response	22	7.30E−05
	GO:0006813 ~ potassium ion transport	9	3.70E−04
	GO:0007267 ~ cell–cell signaling	16	4.01E−04
	GO:0007268 ~ chemical synaptic transmission	14	2.09E−03
	GO:0007166 ~ cell surface receptor signaling pathway	15	2.45E−03
	GO:0034765 ~ regulation of ion transmembrane transport	9	2.68E−03
	GO:0007165 ~ signal transduction	40	3.61E−03
	GO:0006955 ~ immune response	19	4.38E−03
	GO:0007596 ~ blood coagulation	11	6.43E−03
	GO:0055085 ~ transmembrane transport	13	6.55E−03
	GO:0030198 ~ extracellular matrix organization	11	9.83E−03
	GO:0018108 ~ peptidyl-tyrosine phosphorylation	9	1.75E−02
	GO:0006508 ~ proteolysis	19	2.31E−02
	GO:0006810 ~ transport	14	3.77E−02
	GO:0007399 ~ nervous system development	12	4.57E−02
	GO:0006898 ~ receptor-mediated endocytosis	9	4.74E−02
KEGG Pathway	hsa04080:Neuroactive ligand-receptor interaction	17	1.86E−03
	hsa04024:cAMP signaling pathway	13	4.68E−03
	hsa04020:Calcium signaling pathway	12	6.04E−03
	hsa04514:Cell adhesion molecules (CAMs)	10	1.04E−02
	hsa04022:cGMP-PKG signaling pathway	10	1.97E−02
	hsa04614:Renin-angiotensin system	4	2.00E−02
	hsa04964:Proximal tubule bicarbonate reclamation	4	2.00E−02
	hsa04060:Cytokine-cytokine receptor interaction	13	2.16E−02
	hsa04924:Renin secretion	6	2.34E−02
	hsa04261:Adrenergic signaling in cardiomyocytes	9	2.48E−02
	hsa02010:ABC transporters	5	2.54E−02
	hsa04062:Chemokine signaling pathway	10	4.84E−02

### Construction and validation of an eight-gene prognostic model

**Fig. 4:**
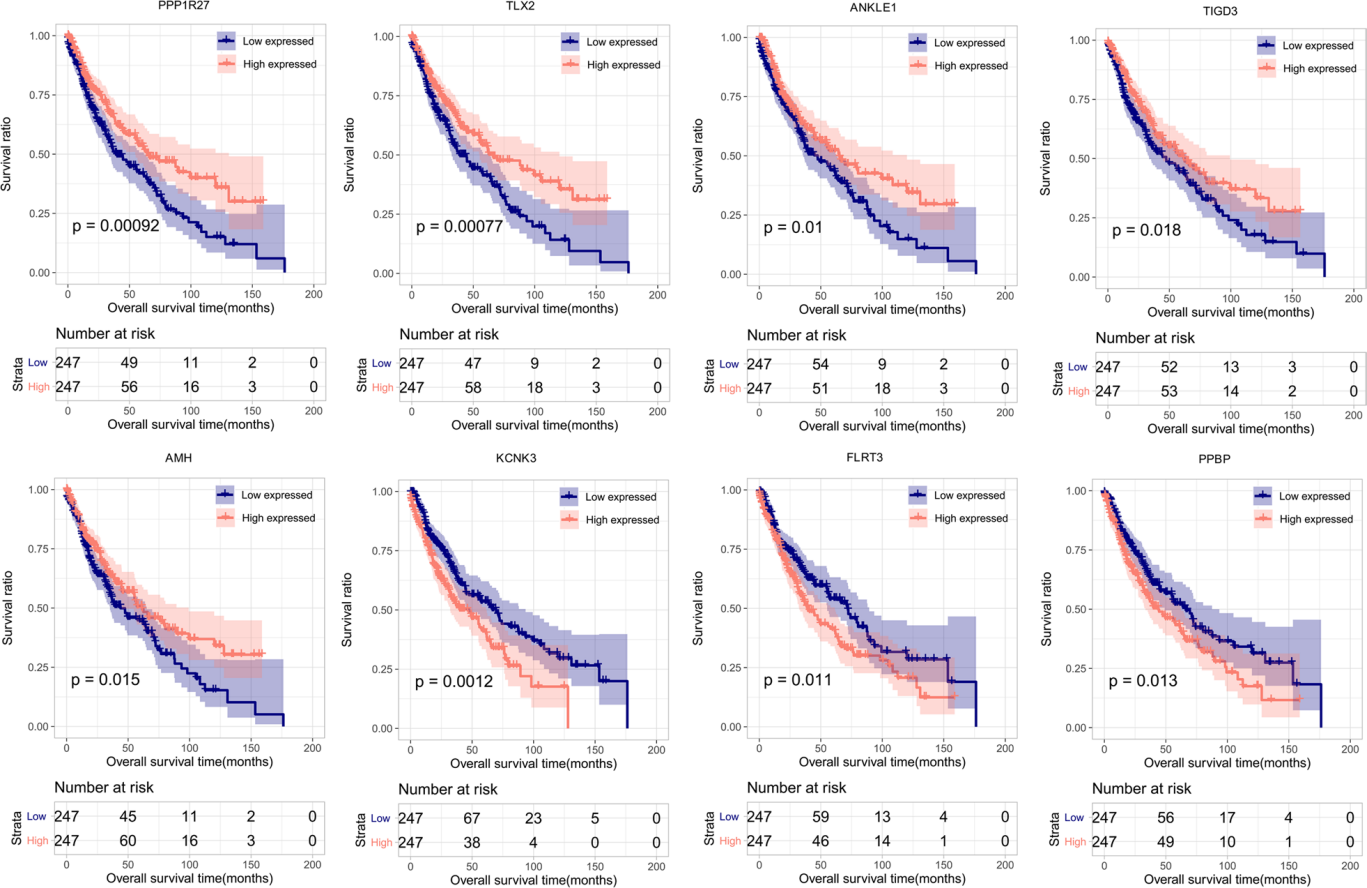
Kaplan–Meier survival curves for high- and low-expressed tumor samples in the TCGA set according to the median expression level of each optimal signature gene

The results of the univariate Cox regression analysis indicate that a total of 77 mRNAsi-related DEGs were significantly associated with prognosis. Applying LASSO Cox regression analysis (1 mean squared error = 0.03871 (Additional file 1: Fig. S1)), we then selected a set of eight prognostic signature genes (PPP1R27, TLX2, ANKLE1, TIGD3, AMH, KCNK3, FLRT3, and PPBP). Based on the median expression level of each optimal signature gene, tumor samples were classified into high- and low-expression groups with significantly different OS times (*p* < 0.05, Fig. 4).

The expression data and the LASSO regression coefficients of the eight signature genes were used to calculate the risk score, as follows:

$$\begin{aligned} {\text{RS}} & = \left( { - 0.0{9567}00{91}} \right)*{\text{Exp}}_{{{\text{PPP1R27}}}} + \left( { - 0.0{3266893}} \right) \\ & \quad *{\text{Exp}}_{{{\text{TLX2}}}}+ \left( { - 0.0{21438984}} \right)*{\text{Exp}}_{{{\text{ANKLE1}}}} \\ & \quad + \left( { - 0.0{24649}0{27}} \right)*{\text{Exp}}_{{{\text{TIGD3}}}}+ \left( { - 0.00{5}0{64291}} \right) \\ & \quad*{\text{Exp}}_{{{\text{AMH}}}} + \left( {0.0{94337731}} \right)*{\text{Exp}}_{{{\text{KCNK3}}}} \\&\quad+ \left( {0.0{1}00{97571}} \right)*{\text{Exp}}_{{{\text{FLRT3}}}} + \left( {0.0{1692}0{132}} \right)\\ &\quad *{\text{Exp}}_{{{\text{PPBP}}}} \end{aligned}$$

**Fig. 5:**
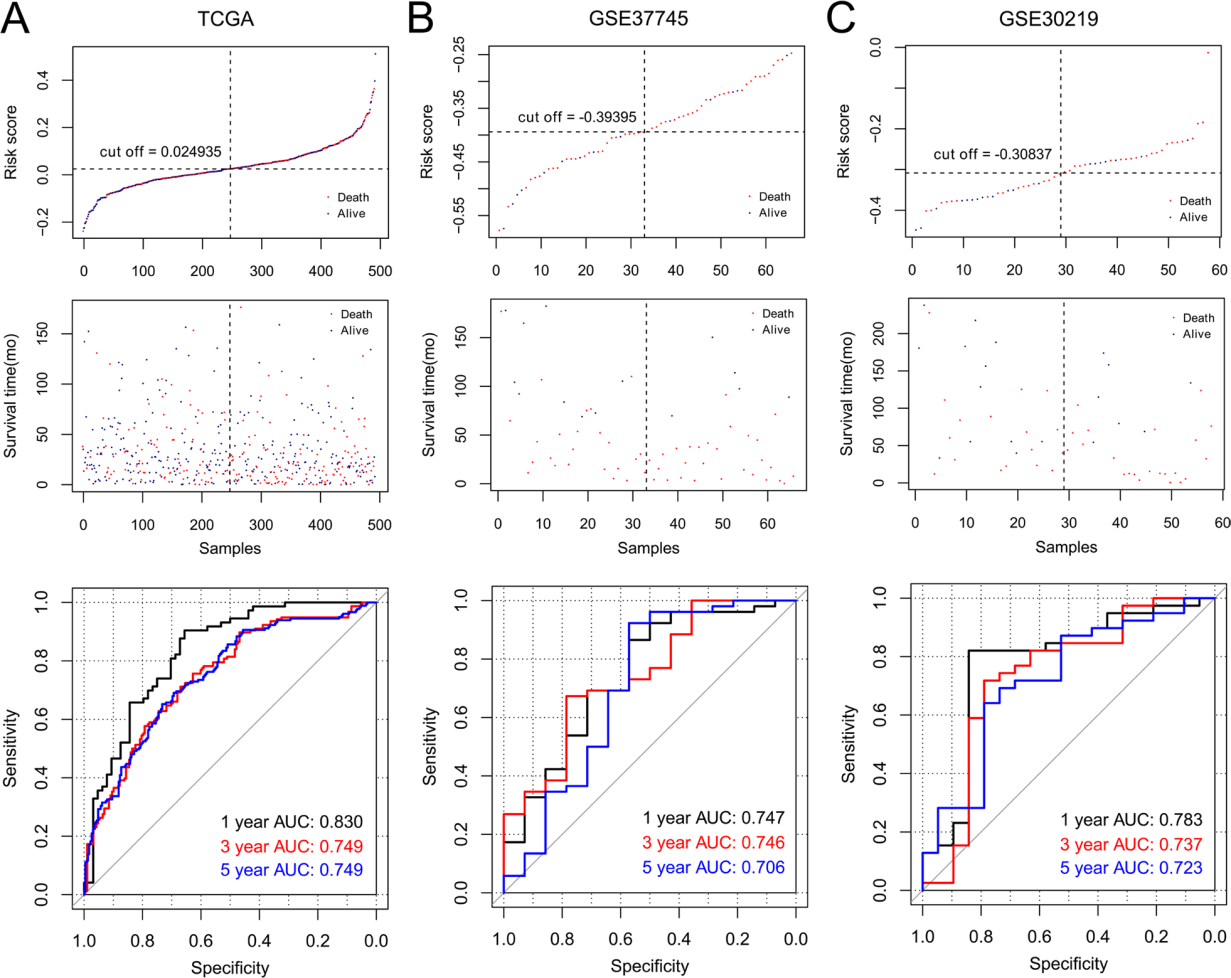
Risk score distribution (*upper*), survival analysis (*middle*), and ROC curve analysis (*lower*) in the TCGA set (**a**), GSE37745 dataset (**b**), and GSE30219 dataset (**c**)

**Fig. 6:**
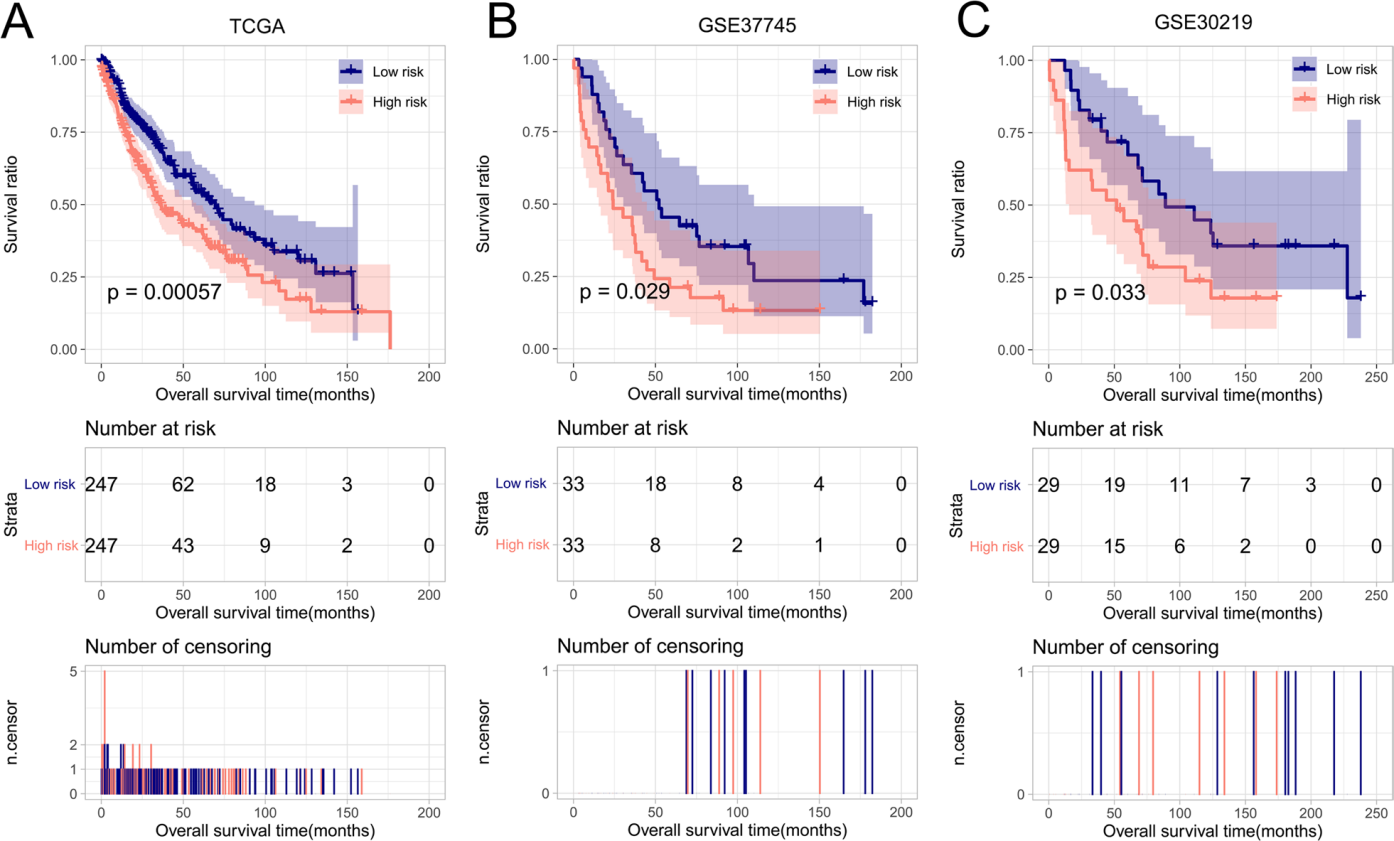
Kaplan–Meier survival curves for high- and low-risk tumor samples in the TCGA set (**a**), GSE37745 dataset (**b**), and GSE30219 dataset (**c**)

The TCGA dataset was consequently dichotomized into high- -and low-risk groups. In the ROC curve analysis, the 1-, 3-, and 5-year AUC values were 0.830, 0.749, and 0.749, respectively (Fig. 5a). The OS time was significantly longer in high-risk patients than in low-risk patients (*p* = 0.00057, Fig. 6a). Furthermore, the eight-gene risk score model was applied to GSE37745 and GSE30219 datasets to validate the predictive performance of the model. The GSE37745 and GSE30219 datasets were divided by the eight-gene risk score into two risk groups with differential survival times (*p* = 0.029, 0.033, Fig. 6b, c; 1-year AUC = 0747, 0.783; 3-year AUC = 0.746, 0.737; 5-year AUC = 0.706, 0.723, Fig. 5b, c).

**Fig. 7:**
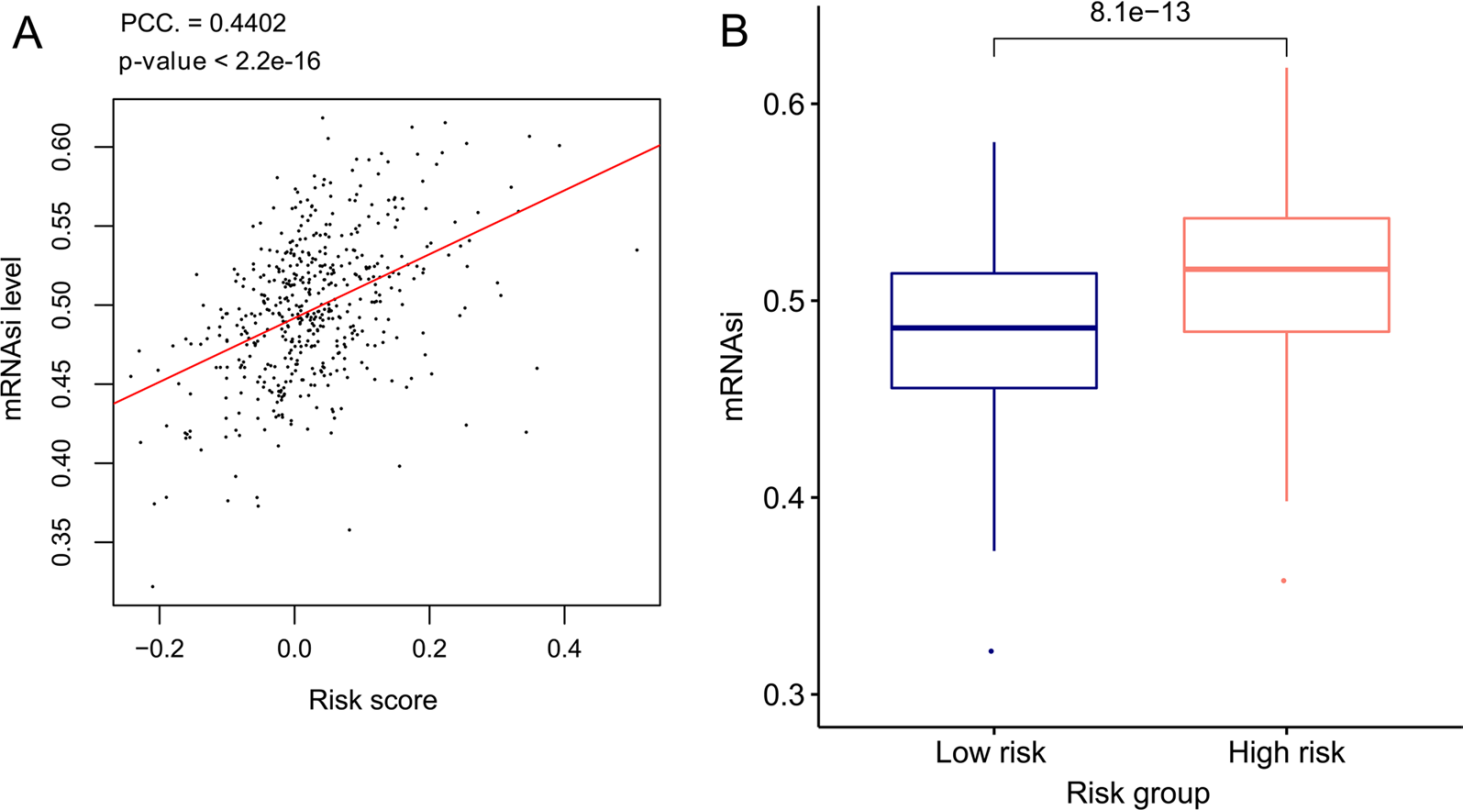
Associations of risk score with stemness index. **a** Correlation of mRNAsi values with risk score; **b** comparison of mRNAsi values between high- and low-risk samples in the TCGA set

In addition, we assessed the associations of mRNAsi levels with the risk score in the TCGA dataset. As shown in Fig. 7, the mRNAsi level was positively correlated with the risk score (PCC = 0.4402, *p*-value = 2.2E−16) and was significantly elevated in low-risk samples compared to high-risk samples (*p*-value = 8.1E−13).

### Identification of independent prognostic factors and stratification analysis

Using the TCGA data, with clinical factors and risk score model status as variables, we performed univariate and multivariate Cox regression analyses to identify prognostic factors. In Table 3, we show that recurrence (HR = 2.047, 95%CI = 1.475–2.843, *p*-value = 1.880E−05) and risk score (HR = 1.571, 95%CI = 1.128–2.186, *p*-value = 7.530E−03) were independent prognostic factors.

**Table 3:** Identification of independent prognostic factors

Clinical characteristics	Uni-variable cox			Multi-variable cox		
	HR	95%CI	*P*-value	HR	95%CI	*P*-value
Age (years, mean ± SD)	1.016	0.999–1.033	5.86E−02	–	–	–
Gender (male/female)	1.196	0.868–1.648	2.73E−01	–	–	–
Pathologic M (M0/M1/-)	3.095	0.985–7.574	9.12E−02	–	–	–
Pathologic N (N0/N1/N2/N3/-)	1.147	0.943–1.395	1.71E−01	–	–	–
Pathologic T (T1/T2/T3/T4)	1.341	1.124–1.600	1.11E−03	1.308	0.989–1.731	5.986E−02
Pathologic stage (I/II/III/IV/-)	1.273	1.079–1.502	4.04E−03	1.009	0.768–1.324	9.494E−01
Radiation therapy (yes/no/-)	1.221	0.794–1.877	3.62E−01	–	–	–
Recurrence (yes/no/-)	2.240	1.625–3.086	4.11E−07	2.047	1.475–2.843	1.880E−05
RS model status (high/low)	1.611	1.225–2.115	5.69E−04	1.571	1.128–2.186	7.530E−03

RS, risk score; HR, hazard ratio; CI, confidence interval

**Fig. 8:**
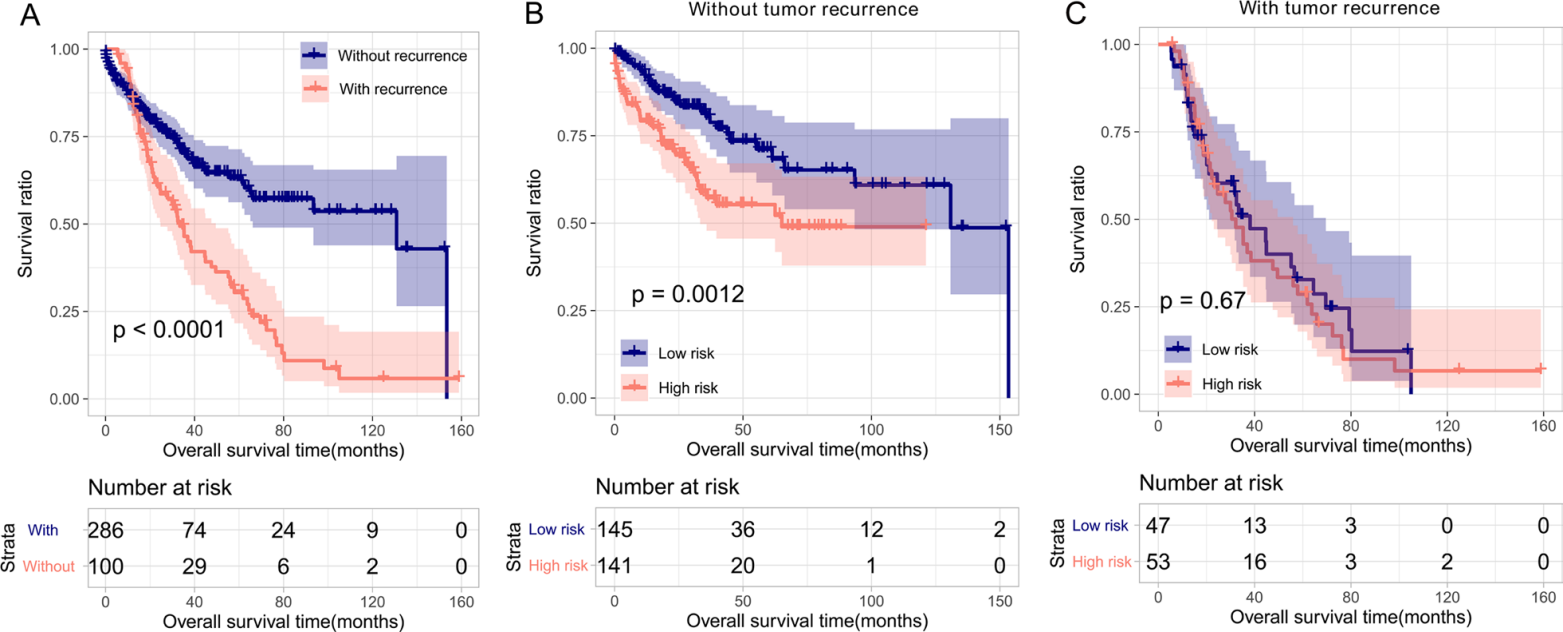
Stratification analysis. **a** Kaplan–Meier survival curves for patients with and without tumor recurrence. **b** Kaplan–Meier survival curves of high- and low-risk patients without tumor recurrence; **c** Kaplan–Meier survival curves of high- and low-risk patients with tumor recurrence

As depicted in Fig. 8a, significantly different OS times were observed between patients with (N = 286) and without recurrence (N = 100, *p* < 0.0001). Stratification analysis was conducted according to recurrence. In patients without tumor recurrence, a worse prognosis was observed in the high-risk subgroup than in the low-risk subgroup (*p* = 0.0012, Fig. 8b). Regarding patients with tumor recurrence, the difference in OS time was insignificant between the two risk subgroups (*p* = 0.67, Fig. 8c).

### The two risk groups had distinct immune characteristics and were significantly involved in DNA-repair-related pathways

**Fig. 9:**
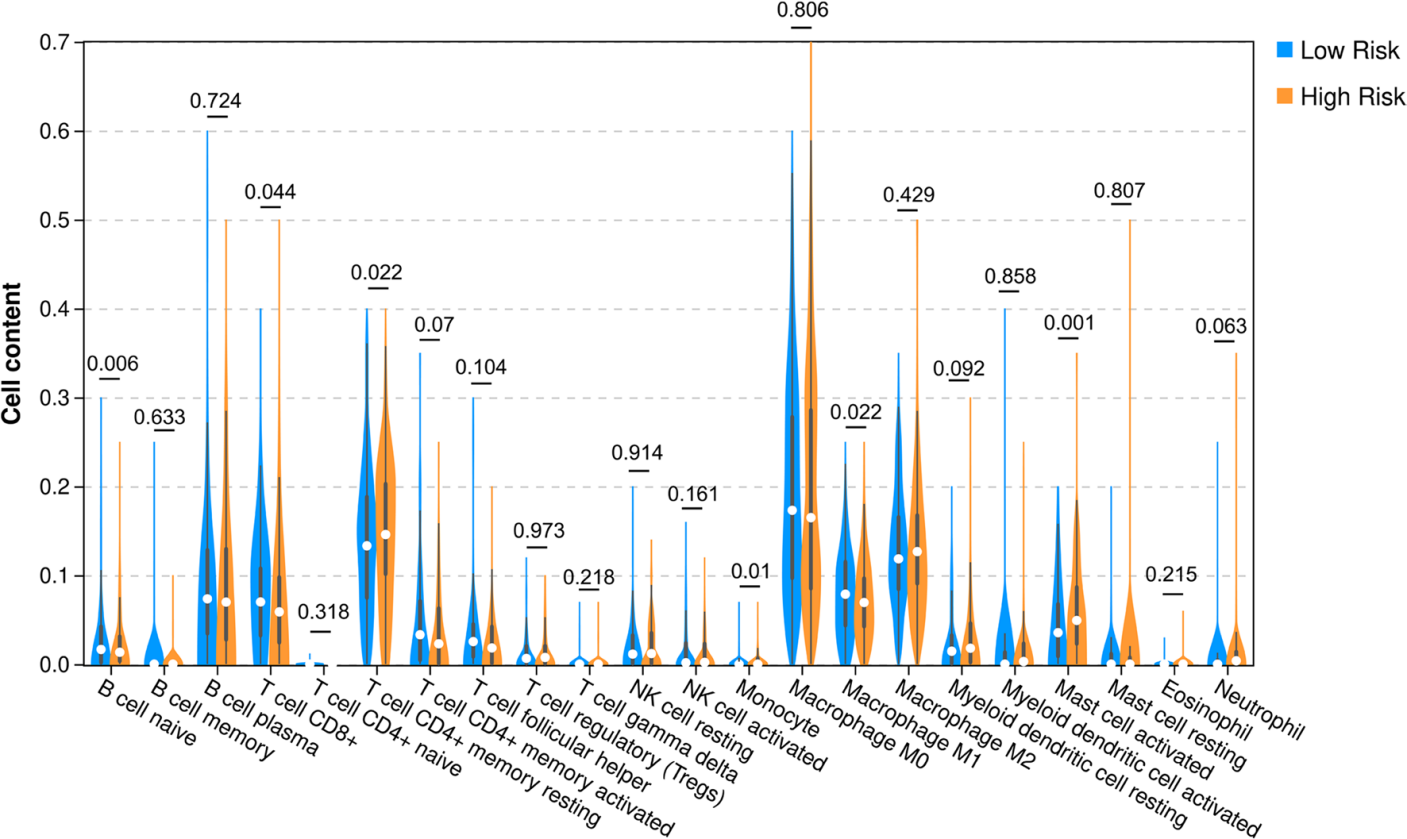
Comparative analysis of fractions of tumor-infiltrating immune cells in high- and low-risk samples of the TCGA set

**Fig. 10:**
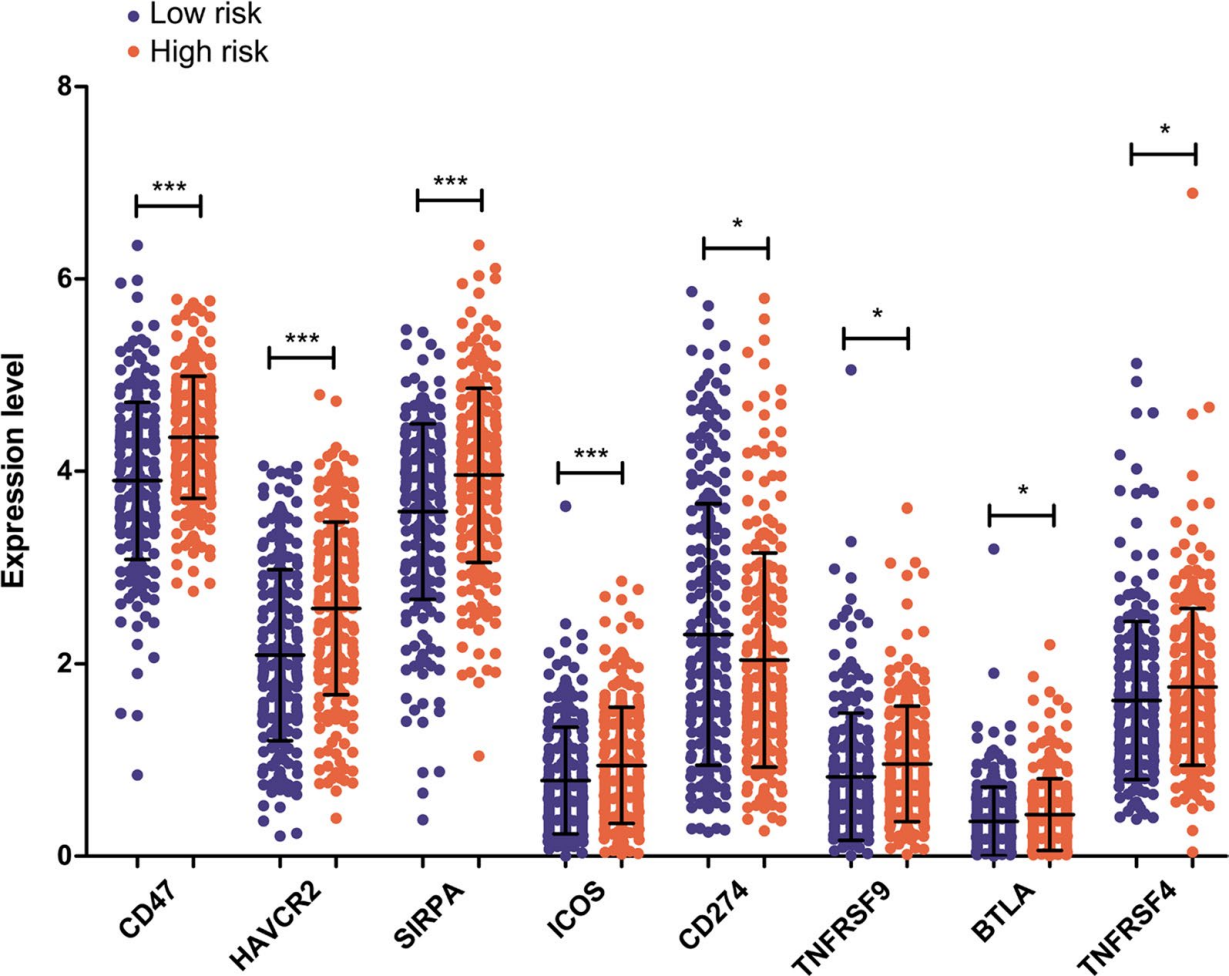
Expression levels of eight differentially expressed immune checkpoint genes in high- and low-risk patients

There is evidence that cancer stemness is associated with immune checkpoint genes and the proportion of TIICs in the tumor microenvironment [22]. Therefore, we assayed the proportion of different types of TIICs. Compared to high-risk samples, low-risk samples had significantly increased percentages of naïve B cells (*p* = 0.006), CD8+ T cells (*p* = 0.044), and M1 macrophages (*p* = 0.022) and decreased percentages of resting memory CD4+ T cells (*p* = 0.022), monocytes (*p* = 0.01), and activated mast cells (*p* = 0.001, Fig. 9). We compared the expression of 18 immune checkpoint genes. Notably, CD47, HAVCR2, SIRPA, ICOS, TNFRSF9, BTLA, and TNFRSF4 were significantly downregulated in low-risk samples compared to high-risk samples (*p* < 0.05, Fig. 10). This result indicates that the two risk samples had different immune characteristics.

**Fig. 11:**
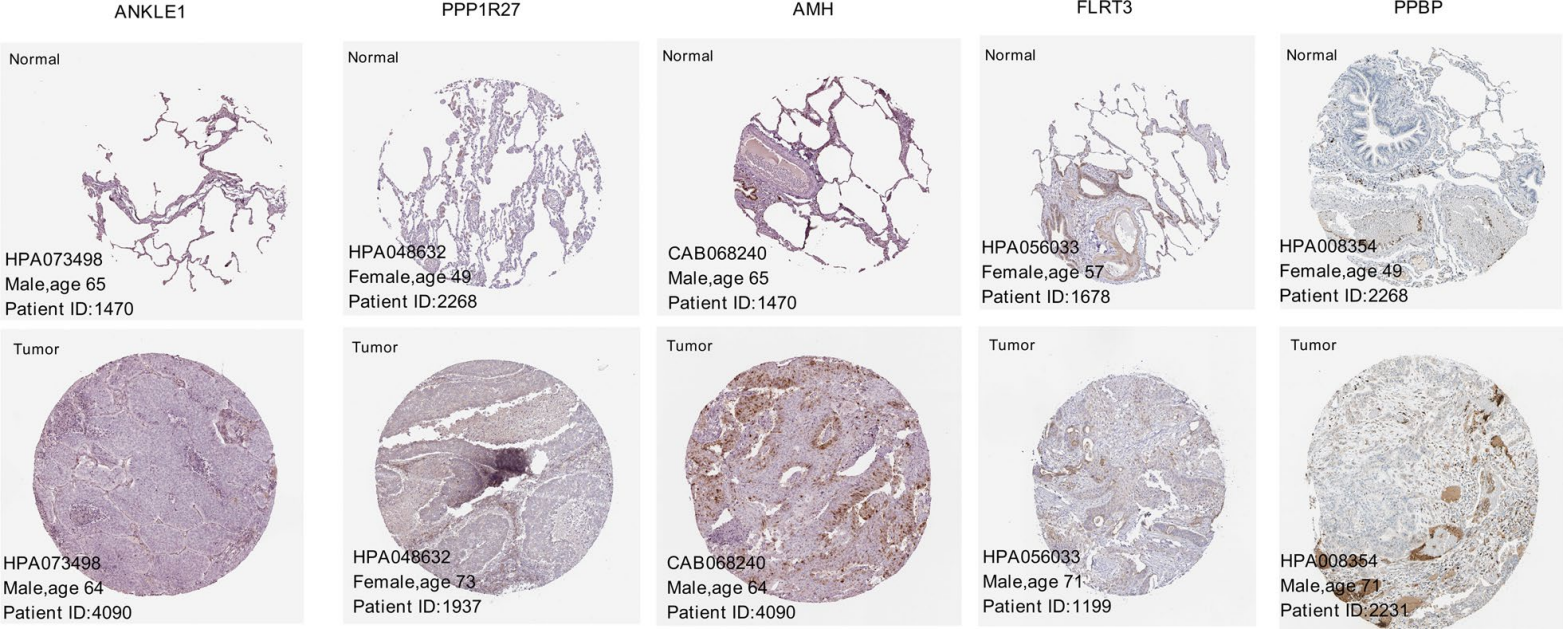
Immunohistochemical staining of ANKLE1, PPP1R27, AMH, FLRT3, and PPBP in normal and tumor tissues

Moreover, we identified nine DNA-repair-related KEGG pathways that were significantly associated with the obtained risk subgroups (FDR < 0.05), including base excision repair, RNA degradation, RNA polymerase, and spliceosome (Table 4). Using data from the HPA database, we found immunohistochemical images of five prognostic signature genes in normal and tumor tissues (Fig. 11), including three upregulated genes (ANKLE1, PPP1R27, and AMH) and two downregulated genes (FLRT3 and PPBP). The immunohistochemical results were consistent with our differential expression analysis (Fig. 11, Additional file 2: Table S1).

**Table 4:** Significant KEGG pathways associated with the obtained risk subgroups

Pathway name	Size	Enrichment score	Normalized enrichment score	FDR (q-value)
KEGG_BASE_EXCISION_REPAIR	33	− 0.642	− 1.896	0
KEGG_CELL_CYCLE	112	− 0.522	− 1.774	1.43E−02
KEGG_DNA_REPLICATION	33	− 0.776	− 1.980	0
KEGG_HOMOLOGOUS_RECOMBINATION	22	− 0.738	− 1.793	1.97E−03
KEGG_MISMATCH_REPAIR	22	− 0.747	− 1.881	0
KEGG_NUCLEOTIDE_EXCISION_REPAIR	43	− 0.621	− 1.978	0
KEGG_RNA_DEGRADATION	48	− 0.516	− 1.760	1.02E−02
KEGG_RNA_POLYMERASE	26	− 0.556	− 1.668	4.74E−02
KEGG_SPLICEOSOME	91	− 0.484	− 1.868	3.85E−02

Size, the count of genes significantly enriched in a pathway. FDR, false discovery rate

## Discussion

Functionally defined by their high tumorigenic potency and self-renewal properties, CSCs are a critical driving force for cancer metastasis, recurrence, and chemoresistance and have been increasingly acknowledged as potential therapeutic targets [29]. In the present study, we focused on the identification of CSC-related prognostic genes for survival prediction in LUSC. By exploiting gene expression data from TCGA, we identified 404 mRNAsi-related DEGs in tumors—77 of which were significantly associated with survival—and constructed a risk score model using eight prognostic genes obtained using LASSO Cox regression analysis. The eight-gene risk score partitioned the TCGA dataset into two risk groups with significantly different OS times (*p* = 0.00057, 1-, 3-, and 5-year AUC = 0.830, 0.749 and 0.749, respectively). The value of mRNAsi was positively correlated with the risk score. The capability of the eight-gene risk score to stratify survival into subgroups was successfully validated in the two validation datasets, as evidenced by the significant log-rank p-values and high AUC values (0.7–0.8). Furthermore, the eight-gene risk score was shown to be an independent prognostic factor, regardless of recurrence rate. These results extend our knowledge of the maintenance and promotion of the malignant characteristics of CSCs and may contribute to a more accurate prognosis (survival prediction) as well as targeted therapies for LUSC.

The eight-gene prognostic signature identified in our study was composed of PPP1R27, TLX2, ANKLE1, TIGD3, AMH, KCNK3, FLRT3, and PPBP. Anti-Mullerian hormone (AMH), a member of the transforming growth factor/bone morphogenetic protein superfamily, participates in regulating epithelial-mesenchymal transition (EMT), epithelial plasticity, and chemoresistance in lung cancer [30]. Moreover, AMH is an immune-related prognostic gene in LUSC and has been used to construct a prognostic model in LUSC [31]. Zhuang et al. also found that AMH is a LUSC-related immune gene and is not associated with distant metastasis [32]. TWIK-related acid-sensitive potassium channel 1 (TASK1), encoded by KCNK3, is associated with pulmonary circulation and controls pulmonary arterial tone, which may contribute to poor prognosis in lung cancer patients [33]. KCNK3 has been shown to influence apoptosis and proliferation in NSCLCs, and KCNK3 knockdown increases apoptosis in tumor cells [34]. Therefore, KCNK3 may play a role in cell motility, activation and proliferation [35]. FLRT3 is a transmembrane protein belonging to the family of axon guidance-related factors. FLRT3, which is found in many tissues and is involved in cell adhesion and adipocytokine signalling pathways [35], has been implicated in the progression and prognosis of LUSC and could serve as a prognostic biomarker [36]. Pro-platelet basic protein (PPBP) and chemokine ligand 7 (CXCL7) are platelet activation markers that act as inducers of macrophage chemotaxis and mediators of neutrophil accumulation [37]. PPBP is a survival-related hub gene in lung adenocarcinoma [18] and non-smoker females with lung cancer [38]. Furthermore, studies have shown that PPBP is significantly increased in lung cancer tissue and blood samples, making it a novel diagnostic biomarker for lung carcinoma [39, 40]. T-cell leukemia homeobox 2 (TLX2) has prognostic value in uterine sarcoma [41]. ANKLE1 (ankyrin repeat and LEM domain) is involved in DNA damage response and DNA repair and is associated with breast cancer development [42, 43]. In addition, ANKLE1 has been repeatedly shown to be involved in DNA repair pathways in preclinical and in vitro screens, including endonuclease activity, proliferation, and drug response in CRISPR screens of cancer cell lines [44–46]. A study of NSCLC showed that ANKLE1 RNAi in combination with paclitaxel increased the efficacy of drug response [47]. Nonetheless, there is little information regarding the biological functions of PPP1R27 and TIGD3 in cancer. In this study, the capability of the eight-gene risk score to stratify survival time was successfully validated using two validation datasets, suggesting that these eight genes may be prognostic biomarkers for LUSC.

The immune environment is predictive of the prognosis of NSCLCs [48]. TTIICs have been implicated extensively in the initiation and progression of LUSC [49]. In the current study, increased numbers of CD8+ T cells and M1 macrophages and significant downregulation of immune checkpoint genes were observed in low-risk patients compared to high-risk patients. These results showed that the high -and low-risk subgroups possessed distinct immune microenvironment characteristics. CD8+ T cells mediate an anti-tumor immune response [50], and M1 macrophages exert pro-inflammatory and anti-tumor actions [51]. Immune checkpoints are critical for immune suppression and evasion in cancers, and immune checkpoint inhibitors represent an efficient therapeutic approach against a wide spectrum of malignancies [52]. Our results suggest that low-risk patients have a stronger antitumor immune function, which protects against LUSC and achieves a significant survival benefit. Through interactions with the tumor immune microenvironment, CSCs facilitate immune evasion and suppress the immune system to promote tumor progression [53]. Taken together, these results indicate that the crosstalk between CSCs and the immune microenvironment may affect the prognosis of LUSC patients, and further studies are needed to validate these results.

This study has some limitations. Firstly, because all data were obtained from the TCGA and GEO databases, selection bias could not be ruled out. Secondly, 501 LUSC tumor samples and 49 normal control samples were downloaded from the TCGA database, considering that such unequal sample distribution (controls being approximately 10% of tumor samples), may amplify the detection of differences. Thus, further validation is required to support the discovery of this research.

## Conclusion

In this study, we constructed and validated a risk score model based on the expression of eight CSC-related DEGs that could effectively predict LUSC outcomes. These eight CSC-related genes may be prognostic biomarkers and potential therapeutic targets for LUSC. Our study sheds light on the prognostic value of cancer stemness-related genes and their underlying mechanisms and may facilitate personalized counselling and treatment of LUSC. Further research is required to confirm and extend these findings.

## Supplementary Information

**Additional file 1: Figure S1.** The results of Lasso Cox regression analysis.

**Additional file 2: Table S1.** The results of differential expression analysis.

## Data Availability

The datasets used and/or analysed during the current study are available from the corresponding author on reasonable request.

## Abbreviations

CSCs: Cancer stem cells
LUSC: Lung squamous cell carcinoma
DEGs: Differentially expressed genes
NSCLC: Non-small cell lung cancer
TCGA: The Cancer Genome Atlas
OS: Overall survival
KM: Kaplan–Meier
GO: Gene ontology
KEGG: Kyoto encyclopedia of genes and genomes
LASSO: Least absolute shrinkage and selection operator
TIICs: Tumor-infiltrating immune cells
HPV: Human protein atlas
OS: Overall survival
EMT: Epithelial-mesenchymal transition
CXCL7: Chemokine ligand 7
TLX2: T cell leukemia homeobox 2

## References

[1] Ferlay J, Colombet M, Soerjomataram I, Mathers C, Parkin DM, Piñeros M, Znaor A, Bray F (2019). Estimating the global cancer incidence and mortality in 2018: GLOBOCAN sources and methods. Int J Cancer.

[2] Dong M, Liu J, Gong H, Li X, Song Z, Zhao H, Wei S, Chen G, Zhou Q, Liu H (2021). The analysis of surgical prognostic factors and molecular typing of locally advanced lung squamous cell carcinomas. Asia Pac J Clin Oncol.

[3] Mederos N, Friedlaender A, Peters S, Addeo A (2020). Gender-specific aspects of epidemiology, molecular genetics and outcome: lung cancer. ESMO open.

[4] Siegel RL, Miller KD, Jemal A (2019). Cancer statistics, 2019. CA Cancer J Clin.

[5] Lei Y, Xiao J, Zhao W, Liu F, Sui Y, Wang K, Liu Y (2022). Myc pathway-guided alternative splicing events predict the overall survival of lung squamous cell carcinoma. All Life.

[6] Chang JC (2016). Cancer stem cells: role in tumor growth, recurrence, metastasis, and treatment resistance. Medicine.

[7] Heng WS, Gosens R, Kruyt FAE (2019). Lung cancer stem cells: origin, features, maintenance mechanisms and therapeutic targeting. Biochem Pharmacol.

[8] Prasad S, Ramachandran S, Gupta N, Kaushik I, Srivastava SK (2020). Cancer cells stemness: a doorstep to targeted therapy. Biochim Biophys Acta.

[9] Saygin C, Matei D, Majeti R, Reizes O, Lathia JD (2019). Targeting cancer stemness in the clinic: from hype to hope. Cell Stem Cell.

[10] Walcher L, Kistenmacher AK, Suo H, Kitte R, Dluczek S, Strauß A, Blaudszun AR, Yevsa T, Fricke S, Kossatz-Boehlert U (2020). Cancer stem cells-origins and biomarkers: perspectives for targeted personalized therapies. Front Immunol.

[11] Barbato L, Bocchetti M, Di Biase A, Regad T (2019). Cancer stem cells and targeting strategies. Cells.

[12] Pirozzi G, Tirino V, Camerlingo R, La Rocca A, Martucci N, Scognamiglio G, Franco R, Cantile M, Normanno N, Rocco G (2013). Prognostic value of cancer stem cells, epithelial-mesenchymal transition and circulating tumor cells in lung cancer. Oncol Rep.

[13] O'Conor CJ, Chen T, González I, Cao D, Peng Y (2018). Cancer stem cells in triple-negative breast cancer: a potential target and prognostic marker. Biomark Med.

[14] Muinao T, Deka Boruah HP, Pal M (2018). Diagnostic and prognostic biomarkers in ovarian cancer and the potential roles of cancer stem cells: an updated review. Exp Cell Res.

[15] Liao Y, Xiao H, Cheng M, Fan X (2020). Bioinformatics analysis reveals biomarkers with cancer stem cell characteristics in lung squamous cell carcinoma. Front Genet.

[16] Qin S, Long X, Zhao Q, Zhao W (2020). Co-expression network analysis identified genes associated with cancer stem cell characteristics in lung squamous cell carcinoma. Cancer Invest.

[17] Giannos P, Kechagias KS, Gal A (2021). Identification of prognostic gene biomarkers in non-small cell lung cancer progression by integrated bioinformatics analysis. Biology (Basel).

[18] Yu Y, Tian X (2020). Analysis of genes associated with prognosis of lung adenocarcinoma based on GEO and TCGA databases. Medicine.

[19] Rousseaux S, Debernardi A, Jacquiau B, Vitte AL, Vesin A, Nagy-Mignotte H, Moro-Sibilot D, Brichon PY, Lantuejoul S, Hainaut P (2013). Ectopic activation of germline and placental genes identifies aggressive metastasis-prone lung cancers. Sci Transl Med.

[20] Jabs V, Edlund K, Knig H, Grinberg M, Micke P (2017). Integrative analysis of genome-wide gene copy number changes and gene expression in non-small cell lung cancer. PLOS ONE.

[21] Lohr M, Hellwig B, Edlund K, Mattsson J, Rahnenführer J (2015). Identification of sample annotation errors in gene expression datasets. Arch Toxicol.

[22] Malta TM, Sokolov A, Gentles AJ, Burzykowski T, Poisson L, Weinstein JN, Kamińska B, Huelsken J, Omberg L, Gevaert O (2018). Machine learning identifies stemness features associated with oncogenic dedifferentiation. Cell.

[23] Ritchie ME, Phipson B, Wu D, Hu Y, Law CW, Shi W, Smyth GK (2015). limma powers differential expression analyses for RNA-sequencing and microarray studies. Nucleic Acids Res.

[24] Sokolov A, Paull EO, Stuart JM (2016). One-class detection of cell states in tumor subtypes. Pac Symp Biocomput.

[25] Liang R, Zhi Y, Zheng G, Zhang B, Zhu H, Wang M (2019). Analysis of long non-coding RNAs in glioblastoma for prognosis prediction using weighted gene co-expression network analysis, Cox regression, and L1-LASSO penalization. Onco Targets Ther.

[26] Chen B, Khodadoust MS, Liu CL, Newman AM, Alizadeh AA (2018). Profiling tumor infiltrating immune cells with CIBERSORT. Methods Mol Biol (Clifton, NJ).

[27] He W, Chen L, Yuan K, Zhou Q, Peng L, Han Y (2018). Gene set enrichment analysis and meta-analysis to identify six key genes regulating and controlling the prognosis of esophageal squamous cell carcinoma. J Thorac Dis.

[28] Miura K, Ishida K, Fujibuchi W, Ito A, Niikura H, Ogawa H, Sasaki I (2012). Differentiating rectal carcinoma by an immunohistological analysis of carcinomas of pelvic organs based on the NCBI Literature Survey and the Human Protein Atlas database. Surg Today.

[29] Huang T, Song X, Xu D, Tiek D, Goenka A, Wu B, Sastry N, Hu B, Cheng SY (2020). Stem cell programs in cancer initiation, progression, and therapy resistance. Theranostics.

[30] Beck TN, Korobeynikov VA, Kudinov AE, Georgopoulos R, Solanki NR, Andrews-Hoke M, Kistner TM, Pépin D, Donahoe PK, Nicolas E (2016). Anti-Müllerian hormone signaling regulates epithelial plasticity and chemoresistance in lung cancer. Cell Rep.

[31] Li R, Liu X, Zhou XJ, Chen X, Li JP, Yin YH, Qu YQ (2020). Identification of a prognostic model based on immune-related genes of lung squamous cell carcinoma. Front Oncol.

[32] Zhuang Y, Li S, Liu C, Li G (2021). Identification of an individualized immune-related prognostic risk score in lung squamous cell cancer. Front Oncol.

[33] Olschewski A, Veale EL, Nagy BM, Nagaraj C, Kwapiszewska G, Antigny F, Lambert M, Humbert M, Czirják G, Enyedi P (2017). TASK-1 (KCNK3) channels in the lung: from cell biology to clinical implications. Eur Respir J.

[34] Leithner K, Hirschmugl B, Li Y, Tang B, Papp R, Nagaraj C, Stacher E, Stiegler P, Lindenmann J, Olschewski A (2016). TASK-1 regulates apoptosis and proliferation in a subset of non-small cell lung cancers. PLOS ONE.

[35] Lacy SE, Bönnemann CG, Buzney EA, Kunkel LM (1999). Identification of FLRT1, FLRT2, and FLRT3: a novel family of transmembrane leucine-rich repeat proteins. Genomics.

[36] Ma X, Ren H, Peng R, Li Y, Ming L (2020). Identification of key genes associated with progression and prognosis for lung squamous cell carcinoma. PeerJ.

[37] Smith NL, Bromley MJ, Denning DW, Simpson A, Bowyer P (2015). Elevated levels of the neutrophil chemoattractant pro-platelet basic protein in macrophages from individuals with chronic and allergic aspergillosis. J Infect Dis.

[38] Shi K, Li N, Yang M, Li W (2019). Identification of key genes and pathways in female lung cancer patients who never smoked by a bioinformatics analysis. J Cancer.

[39] Xiong D, Pan J, Zhang Q, Szabo E, Miller MS, Lubet RA, You M, Wang Y (2017). Bronchial airway gene expression signatures in mouse lung squamous cell carcinoma and their modulation by cancer chemopreventive agents. Oncotarget.

[40] Ulivi P, Mercatali L, Casoni GL, Scarpi E, Bucchi L, Silvestrini R, Sanna S, Monteverde M, Amadori D, Poletti V (2013). Multiple marker detection in peripheral blood for NSCLC diagnosis. PLOS ONE.

[41] Zhou JG, Zhao HT, Jin SH, Tian X, Ma H (2019). Identification of a RNA-seq-based signature to improve prognostics for uterine sarcoma. Gynecol Oncol.

[42] Kuppuswamy U, Ananthasubramanian S, Wang Y, Balakrishnan N, Ganapathiraju MK (2014). Predicting gene ontology annotations of orphan GWAS genes using protein-protein interactions. Algorithms Mol Biol AMB.

[43] Bakshi D, Katoch A, Chakraborty S, Shah R, Sharma B, Bhat A, Verma S, Bhat GR, Nagpal A, Vaishnavi S (2020). ANKLE1 as new hotspot mutation for breast cancer in indian population and has a role in DNA damage and repair in mammalian cells. Front Genet.

[44] Brachner A, Braun J, Ghodgaonkar M, Castor D, Zlopasa L, Ehrlich V, Jiricny J, Gotzmann J, Knasmuller S, Foisner R (2012). The endonuclease Ankle1 requires its LEM and GIY-YIG motifs for DNA cleavage in vivo. J Cell Sci.

[45] Kabir S, Cidado J, Andersen C, Dick C, Lin PC, Mitros T, Ma H, Baik SH, Belmonte MA, Drew L (2019). The CUL5 ubiquitin ligase complex mediates resistance to CDK9 and MCL1 inhibitors in lung cancer cells. Elife.

[46] Toledo CM, Ding Y, Hoellerbauer P, Davis RJ, Basom R, Girard EJ, Lee E, Corrin P, Hart T, Bolouri H (2015). Genome-wide CRISPR-Cas9 screens reveal loss of redundancy between PKMYT1 and WEE1 in glioblastoma stem-like cells. Cell Rep.

[47] Whitehurst AW, Bodemann BO, Cardenas J, Ferguson D, Girard L, Peyton M, Minna JD, Michnoff C, Hao W, Roth MG (2007). Synthetic lethal screen identification of chemosensitizer loci in cancer cells. Nature.

[48] Öjlert ÅK, Halvorsen AR, Nebdal D, Lund-Iversen M, Solberg S, Brustugun OT, Lingjaerde OC, Helland Å (2019). The immune microenvironment in non-small cell lung cancer is predictive of prognosis after surgery. Mol Oncol.

[49] Zhao J, Bao W, Cai W (2020). Immune infiltration landscape in lung squamous cell carcinoma implications. Biomed Res Int.

[50] Farhood B, Najafi M, Mortezaee K (2019). CD8+ cytotoxic T lymphocytes in cancer immunotherapy: a review. J Cell Physiol.

[51] Najafi M, Hashemi Goradel N, Farhood B, Salehi E, Nashtaei MS, Khanlarkhani N, Khezri Z, Majidpoor J, Abouzaripour M, Habibi M (2019). Macrophage polarity in cancer: a review. J Cell Biochem.

[52] Li B, Chan HL, Chen P (2019). Immune checkpoint inhibitors: basics and challenges. Curr Med Chem.

[53] Silver DJ, Sinyuk M, Vogelbaum MA, Ahluwalia MS, Lathia JD (2016). The intersection of cancer, cancer stem cells, and the immune system: therapeutic opportunities. Neuro Oncol.

