# Supplementary figures and images for: Characterization of a prognostic model for lung squamous cell carcinoma based on eight stemness index-related genes
Source: BMC Pulm Med. 2022 Jun 8;22:224. doi: 10.1186/s12890-022-02011-0 (PMC9178800; doi:10.1186/s12890-022-02011-0)

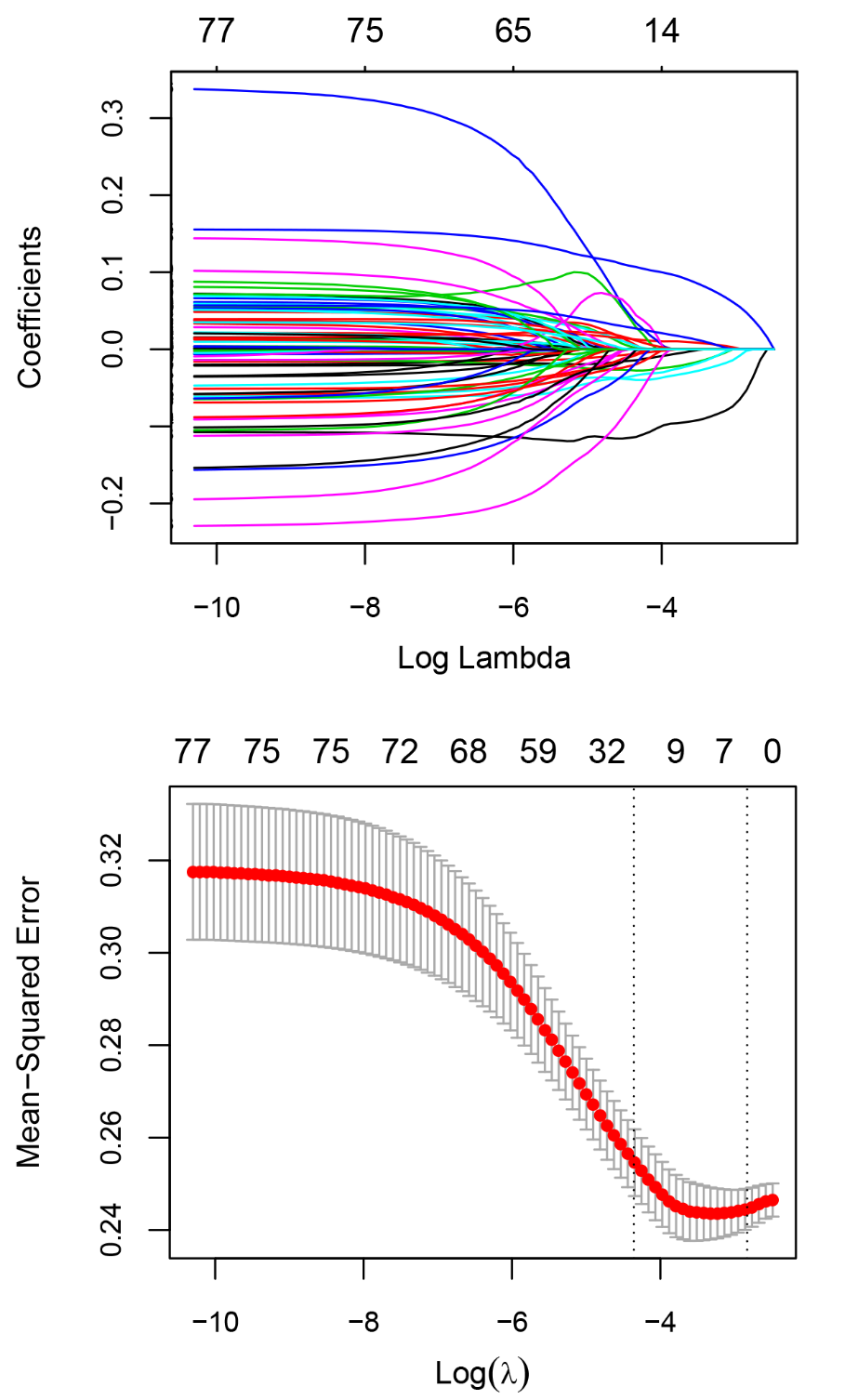


Figure S1. The results of Lasso Cox regression analysis

Supplement: Supplementary file 1 — Additional file 1: Figure S1. The results of Lasso Cox regression analysis. [file 12890_2022_2011_MOESM1_ESM.docx]
